# Quasi-Poisson versus negative binomial regression models in identifying factors affecting initial CD4 cell count change due to antiretroviral therapy administered to HIV-positive adults in North–West Ethiopia (Amhara region)

**DOI:** 10.1186/s12981-016-0119-6

**Published:** 2016-11-09

**Authors:** Awoke Seyoum, Principal Ndlovu, Temesgen Zewotir

**Affiliations:** 1 Department of Statistics, Bahir Dar University, Bahir Dar, Ethiopia; 2 Department of Statistics, University of South Africa, Pretoria, South Africa; 3 School of Mathematics, Statistics and Computer Science, University of KwaZulu Natal, Durban, South Africa

**Keywords:** CD4 cell count change, Longevity, HAART, Quasi-Poisson regression, Negative binomial regression

## Abstract

**Background:**

CD4 cells are a type of white blood cells that plays a significant role in protecting humans from infectious diseases. Lack of information on associated factors on CD4 cell count reduction is an obstacle for improvement of cells in HIV positive adults. Therefore, the main objective of this study was to investigate baseline factors that could affect initial CD4 cell count change after highly active antiretroviral therapy had been given to adult patients in North West Ethiopia.

**Methods:**

A retrospective cross-sectional study was conducted among 792 HIV positive adult patients who already started antiretroviral therapy for 1 month of therapy. A Chi square test of association was used to assess of predictor covariates on the variable of interest. Data was secondary source and modeled using generalized linear models, especially Quasi-Poisson regression.

**Results:**

The patients’ CD4 cell count changed within a month ranged from 0 to 109 cells/mm^3^ with a mean of 15.9 cells/mm^3^ and standard deviation 18.44 cells/mm^3^. The first month CD4 cell count change was significantly affected by poor adherence to highly active antiretroviral therapy (aRR = 0.506, P value = 2e^−16^), fair adherence (aRR = 0.592, P value = 0.0120), initial CD4 cell count (aRR = 1.0212, P value = 1.54e^−15^), low household income (aRR = 0.63, P value = 0.671e^−14^), middle income (aRR = 0.74, P value = 0.629e^−12^), patients without cell phone (aRR = 0.67, P value = 0.615e^−16^), WHO stage 2 (aRR = 0.91, P value = 0.0078), WHO stage 3 (aRR = 0.91, P value = 0.0058), WHO stage 4 (0876, P value = 0.0214), age (aRR = 0.987, P value = 0.000) and weight (aRR = 1.0216, P value = 3.98e^−14^).

**Conclusions:**

Adherence to antiretroviral therapy, initial CD4 cell count, household income, WHO stages, age, weight and owner of cell phone played a major role for the variation of CD4 cell count in our data. Hence, we recommend a close follow-up of patients to adhere the prescribed medication for achievements of CD4 cell count change progression.

## Background

Globally, about 330,000 children were infected with HIV in 2011, and 90% of these infections occurred in Sub-Saharan Africa mainly through mother to child transmission [1]. About 38.1 million people were infected by HIV virus in the world at the end of 2014 and about 25.3 million people died with AIDs related illness [2]. In 2014, about 39.9 million people were living with HIV and the global prevalence rate was 0.8% [3]. In 2009 alone, an estimated 1.3 million adults and children died because of HIV/AIDs in Sub-Saharan African [4]. Most of the people living with HIV/AIDS in Africa are between age 15 and 49, which is the prime age of working [5]. Furthermore, the International Labor Organization (ILO) indicated that in 2005 an estimated number of 2 million workers were unable to work in Africa due to HIV/AIDs illness; and this figure was doubled in 2015 [6]. During the period, around 25.8 million people were living with HIV virus in Sub-Saharan Africa, accounting for 67.7% of the global total [6]. The impact of HIV/AIDs in Africa, on the workforce, increases expenditure on the one hand and decreases productivity on the other [6]. In Ethiopia, about 730,000 people were living with HIV and among these 23,000 died due to AIDs. An estimated prevalence among pregnant women was 1.2%, and one of every 3 children born to these women got infected with HIV [7]. In Amhara Region, all HIV prevalence was estimated to be 1.6% [8] and the prevalence among women attending prenatal clinics from 1999 to 2000 was more than 18% [9]. Therefore, the Amhara region is among the regions that require special attention to HIV- related problems such as recovery of CD4 cell count to highly active antiretroviral therapy (HAART) [10].

Although the current HIV/AIDs surveillance estimates indicate some encouraging signs that the epidemic is stabilizing, the observed changes are not sufficient enough to be compared to the desired goals of response against the epidemic [11]. Availability of information about factors that affect CD4 cell count in the study area at initial stage of treatment is important for HIV patients to have long life period [12]. Information on the rate of initial HAART regimen change and its predictor in Ethiopia is scarce [13, 14]. There is a limited data regarding factors that predict initial CD4 cell count change to HAART medication in the study area [14]. In particular, there are no studies that examine how patient-related factors relate to each other (interact) and their subsequent influence on initial CD4 cell count change [15]. The purpose of this study is thus to identify whether or not specific clinical and socio-demographic factors present at the baseline influence first month CD4 cell count change among HIV positive adults in Amhara region (North west Ethiopia) [16]. Therefore, the present study emphasizes the role of covariates (predictors) that are thought to affect the parameters of the conditional distribution of events, given the covariates. The knowledge and understanding of such factors is important given the increasing number of patients enrolled in HAART [16]. This improvement helps to reduce dropout patients from the treatment. The results of this research can further be used to shape communication and counseling prior to treatment initiation.

## Methods

### Study materials and setting

The data for this study consisted of secondary data, records of social, demographic and clinical characteristics of 792 adult HIV patients recorded after 1 month of therapy by HIV care providers. A Chi square test of association was used to assess predictors of the response variable. The study was cross-sectional, targeted for 6036 HIV/AIDS patients who visited Felege-Hiwot Referral and Teaching Hospital and Health Research center in Bahir Dar, Ethiopia, under the follow-up of ART from September 2005 to August 2012.

### Inclusion criteria

Adult patients, whose ages were 15+ years, with a CD4 cell count below 200 cells/mm^3^ or patients with World Health Organization (WHO) stage IV of HIV disease regardless of CD4 cell count, enrolled at Felege-Hiwot Referral and Teaching Hospital were included under this study.

### Sample size and sampling technique

Out of the targeted HIV/AIDS patients, 792 were selected using stratified random sampling technique considering their residence area as strata using 95% level of confidence and 5% marginal error.

### Data collection tools and procedures

The available information was first observed and discussed with health care service providers at ART section from the hospital. Data was extracted using data extraction format developed by the investigators in consultation with health service providers. All relevant information was collected by health care service providers after theoretical and practical orientations. Charts of patients were retrieved using the patients’ registration card number which was found in the electronic database system.

### Data quality

The quality of the data was controlled by data controllers from the ART section as well as the regional health research center who had intensive ART training from the Ministry of Health for these and other purposes. Data collectors got introductions about definitions of variables in the questionnaires. The data extraction tools and variables included in the analysis were pre-tested for consistency of understanding, review of tools and completeness of data items on 45 random charts. Based on the pilot data result, the necessary amendments were made on the final data extraction format. The retrieval process was closely monitored by the principal investigator throughout the data collection period. Both predictor and response variables were checked regularly for completeness of information. Any problem traced was immediately communicated to data collectors for giving corrections.

### Variable of interest

The variable of interest for this study was CD4 cell count change per mm^3^. The response variable was count data.

### Independent variable

The potential predictor variables for this study were age in years, weight in kg, baseline CD4 cell count, gender (male, female), educational status (no education, primary, secondary and tertiary), disease disclosure (disclosed their disease to family members, closed the disease to family members), residential area (rural, urban), WHO stages (stage 1, stage 2, stage 3 and stage 4), adherence to HAART (poor, fair and good), level of income (low, middle and high), marital status (living with partner, living without partner), and owner of cell phone (with cell phone, without cell phone).

The standard model for count data is Poisson distribution. It is, therefore, useful at the outset to review some fundamental properties and characterize results of the Poisson distribution. If the discrete random variable Y has Poisson distribution with intensity or rate parameter *μ*, *μ* > 0 and t is the exposure defined as the length of time which the event recorded, then Y has the density [17]

1 $${ \Pr }\left( {{\text{Y}} = {\text{y}}} \right)\, = \, \frac{{ e^{ - \mu t} (\mu t)^{y} }}{y!},\quad {\text{y}}\,{ = }\,0, 1, \ldots$$

where $E\left( y \right)\, = \,var\left( y \right)\, = \,\mu t$. If the time period equals to unity, then its density given in (1) equals

2 $${ \Pr }\left( {{\text{Y}} = {\text{y}}} \right)\, = \,\frac{{\varvec{ e}^{{\varvec{ - \mu }}} \varvec{(\mu )}^{\varvec{y}} }}{{\varvec{y!}}}\varvec{,}\quad {\text{y}}\, = \,0, 1, \ldots$$

Equality of mean and variance of Poisson distribution is referred to as the equi-dispersion property of Poisson which is mostly violated in real life data [18].

In generalized linear models, the method of maximum likelihood estimation is usually used to estimate the parameters in the given model [19]. To define likelihood, we have to specify the form of distribution of observation; while to define quasi-likelihood function, we need to specify only the mean–variance relationship and then apply quasi-likelihood for parameter estimation [20]. The important motivation of Poisson distribution from estimation point of view depends on mean–variance relation [20]. In over-dispersed Poisson model, an extra parameter is included which estimates how much larger the variance is than the mean [21]. This parameter estimate is then, used to correct the effects of the larger variance on the P values [22]. In the over-dispersed distribution, one alternative approach to fit extra dispersion parameter which accounts for that extra variance is a Quasi-Poisson model. It has two parameters, namely mean, *μ* and over-dispersion parameter *θ* such that variance is a linear function of mean [23]. Hence for random variable y that follows Quasi-Poisson distribution, we have

3 $$\begin{aligned} E\left( y \right) & = \mu \,{\text{and}} \hfill \\ var\left( y \right)\, & = \,\emptyset\, E(Y)\, = \,\emptyset \,\mu \hfill \\ \end{aligned}$$

for $\emptyset \, > \,1$, we have over-dispersion relative to Poisson. Applying iteratively re-weighted least squares in the more general case involves working with weights say $W^{*} \, = \,\frac{\mu }{ \emptyset }$. This implies that when variance is proportional to mean (not necessarily equal to mean), Poisson estimator is maximum Quasi-Poisson likelihood estimator and the model is said to Quasi-Poisson regression model [21]. The quasi-likelihood function ${\text{K}}(y_{i} ,\,\mu_{i} )$ for each independent observation, $y_{i}$ is defined as

4 $$\frac{{\partial {\text{K }}(y_{i} ,\,\mu_{i} ) }}{{\partial \mu_{i} }}\, = \,\frac{{y_{i} - \mu_{i} }}{{V(\mu_{i} )}}$$

where V is some known function and suppose the expectation, $\mu_{i}$ is some function of parameters *β*_*i*_. Another alternative for modeling over-dispersion is a negative binomial regression model [24] with two parameters and having a form of the Poisson distribution in which the distribution’s parameter itself is considered as random variable. The first two moments of negative binomial regression model are [24].

5 $$\begin{aligned} {\text{Mean}},\,E\left( y \right)\, & = \,\mu \,{\text{and}} \hfill \\ {\text{Variance}},\,var\left( y \right)\, & = \,\mu \,( 1+ \theta \,\mu ) \hfill \\ \end{aligned}$$

If *θ* = 0, there will be no unobserved heterogeneity which results in Poisson variance (Poison model is a special case of negative binomial when *θ* = 0); and if *θ* > 0, variance will be greater than mean and becomes over-dispersed [17]. Using weighted least squares; these models have a little difference with weight-mean relation as shown below [20]:

6 $$\begin{aligned} {\text{W}}\, & = \,{\text{diag }}\left( {\frac{{\mu_{1} }}{\theta },\frac{{\mu_{2} }}{\theta }, \cdots \frac{{\mu_{n} }}{\theta }} \right)\quad {\text{for Quasi-Poisson and}} \hfill \\ {\text{W}}\, & = \,{\text{diag }}\left(\frac{{\mu_{1} }}{{1 + k\mu_{1} }},\frac{{\mu_{2} }}{{1 + k\mu_{2} }}, \cdots \frac{{\mu_{n} }}{{1 + k\mu_{n} }}\right) \\ & \qquad {\text{for Negative Binomial}} \hfill \\ \end{aligned}$$

provided all other elements are zero. The mean-weight relation that exists in model Eq. (6) provides us with full comparison between Quasi-Poisson and negative binomial models where Quasi-Poisson weights are directly proportional to the mean and have concave relation to the mean of negative binomial [20].

**Fig. 1:**
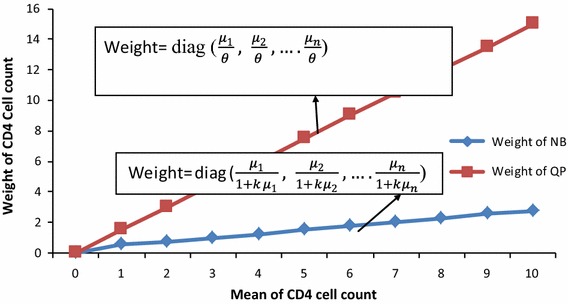
Mean-weight relationship for Quasi-Poisson and negative binomial models

Therefore, the two models, Quasi-Poisson and negative binomial regression models; are to be considered as potential candidates for fitting over-dispersed data. Different scholars such as Ver Hoef [20], Gardner [23], Power [25] and Potts [26] gave different decisions and comments at different times about the models appropriate to over-dispersed data. Therefore, we compared the two models using the following two approaches; comparing the values of log-likelihood, AIC and BIC to assess goodness-of-fit based on our data for the two models as shown in Table 2 [27]; and using mean–variance and mean-weight relation and finding the cut-off- point (boundary value) where the two curves cross each other as shown in Eqs. (3), (5), (6) and (refer Fig. 1). To do this, one can equate the two mean–variance relation equations of the two models (3) and (5) after predicting over-dispersed parameters for the two models separately. Then, one can find the mean value that makes the two graphs cross each other. We consider this value as cut-off point or boundary value. If the mean of response variable (CD4 cell count for our case) is less than the cut-off point, we have to consider negative binomial; while if the mean of the variable of interest is greater than the cut-off point, we need to consider a Quasi-Poisson model [20] (refer to Fig. 1).

### Data analysis

The variables under study were summarized using descriptive statistics such as median for continuous variable and proportions for categorical variables. The data was also analyzed using generalized linear models using Quasi-Poisson regression model. The mean–variance relation, information criteria and the value of Chi square divided by its degree of freedom were used to select the model that fits the data appropriately. Change of deviance was used to measure the extent to which the fit of the model was improved when extra variables were added to the model. The main effects and combination of two ways interaction were fitted, provided that attention was given to hierarchical principle of model fitting. The mean–variance relations for negative binomial and Quasi-Poisson were solved simultaneously to get the value (cut-off points) where the two curves meet each other. The mean of response variable and cut-off points were compared to each other for the two models to select the one which had smaller variation for response variable. The model selected for analysis was the one with smallest information criteria and smallest dispersion parameter and its goodness-of-fit was assessed using Hosmer–Lemeshow goodness-of-fit statistic [28]. Influential observations were identified using cook’s distance against observations [29]. Finally, the linear predictor and its square on the response variable were important for checking appropriateness of link function for the selected model [28]. Data analysis was conducted using SPSS version 21 and R version 3.2.3.

## Results

In Table 1, out of the sample of 792 patients, 40.9% were from rural areas while 59.1% were residing in urban areas; 50.6% were female and 49.4% were male and 44.8% were living with their partners and 55.2% were living without partners. About 47.3% of them disclosed their disease to family members and the rest did not. Of these patients, 46.1% owned cell phone. Lastly 25.5, 44.3 and 30.2% of the patients had good, fair and poor adherence, respectively.

**Table 1:** Baseline socio-demographic and clinical characteristics of the HAART patients (n = 792)

Characteristics	Median	n (%)
Base line weight in kg	62 (58–70)	
Baseline CD cell count	134 (113–180)	
Age in years	36 (28–48)	
First month CD4 cell count change	7 (5–24)	
Gender		
Male		391 (49.4)
Female		401 (50.6)
Educational background		
No educ		160 (20.2)
Primary		205 (25.9)
Secondary		273 (34.5)
Tertiary		154 (19.4)
Residence area		
Urban		468 (59.1)
Rural		324 (40.1)
Marital status		
Living with partner		355 (44.8)
Living without partner		437 (55.2)
Contribution to household income		
Low income		355 (44.8)
Middle income		346 (43.7)
High income		91 (11.5)
WHO stage of HIV stage		
Stage I		101 (12.8)
Stage II		258 (32.6)
Stage III		199 (25.1)
Stage IV		234 (29.5)
Whether or not the patient disclosed the disease		
Disclosed to family members		375 (47.3)
Closed the disease to family members		417 (52.7)
Owner of cell phone		
Yes		365 (46.1)
No		427 (53.9)
First month HAART adherence		
Poor		239 (30.2)
Fair		351 (44.3)
Good		202 (25.5)

**Fig. 2:**
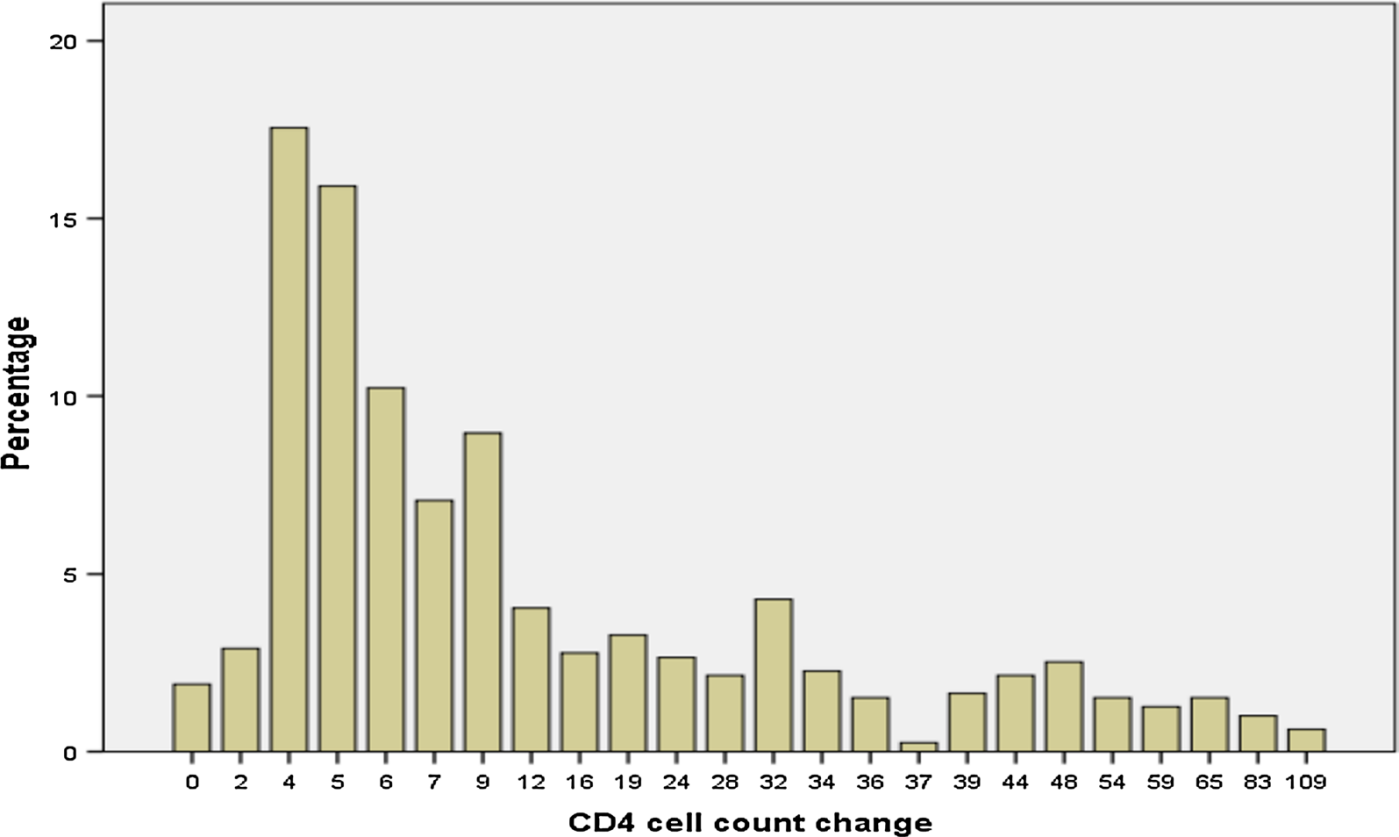
Monthly distribution of changes in CD4 cell count after 1 month of treatment

After 1 month of treatment, the change in CD4 cell count ranged from 0 to 109 cells/mm^3^ with mean 15.9, standard deviation 18. 44 and median 7 cell/mm^3^ (see Fig. 2). Figure 2 also shows that 17.55% of the patients had 4 CD4 cells/mm^3^ and only 0.63% had 109 CD4 cells/mm^3^, and the distribution indicated that variance is about 21 times the mean and this is an indicator of over-dispersed distribution. Using Pearson’s Chi square statistic, deviance divided by degree of freedom, the over-dispersion parameter for Quasi Poisson was $\hat{\emptyset }\, = \,1.49$, which showed that the variance is 49% larger than mean [20]. Using these estimated values, Eqs. (3) and (5) for our data, mean value (cut-off point) which made over-dispersion for Quasi-Poisson [17] and negative-binomial [24] equal to each other was *μ* = 10.5 cells/mm^3^ which is less than the mean of CD4 cell count change (15.9 cells/mm^3^) for our analysis. Therefore, based on the selection criterion, the Quasi-Poisson was selected to fit our data [20]. The two models were also compared using information criteria such as Akakai and Bayesian information criterion [30], and the result is given in Table 2.

**Table 2:** Comparison of Quasi-Poisson and negative binomial using information criteria

Criteria	Quasi-Poisson			Negative-binomial		
	Value	d.f	Vale/d.f.	Value	d.f	Vale/d.f.
Deviance	1090.457	73	1.411	869.625	73	1.128
Pearson Chi-square	1152.646	73	1.491	926.054	73	1.198
Log likelihood	−2158.747			−2657.877		
AIC	4355.493			5353.755		
BIC	4444.310			5442.571		

From Table 2, we observed that deviance was less than Pearson Chi square for both models, but AIC and BIC were smaller for Quasi-Poisson which indicated that Quasi-Poisson was preferable. Hence parameter estimation and identification of predictors of initial CD4 cell count should be conducted using the selected model (Quasi-Poisson model).

From Table 3, considering adherence as a predictor variable, compared to good adherence, log of the expected CD4 cell count change difference between poor adherent patients and good adherent patients was about −0.68 cells/mm^3^, and the difference between fair adherent and good adherent patients was −0.525 cells/mm^3^ per month. In other words, the CD4 cell count change for poor adherents was 0.51 times that of good adherent patients (aRR = 0.51, P value = 2e^−16^). And the rate of change of CD4 cell count for fair adherent patients was 0.59 times (aRR = 0.59, P value = 0.0120) that of good adherent patients keeping the other variables constant. For one year increase of the age of a patient, the log of expected CD4 cell count change decreased by 0.012 cells/mm^3^ (aRR = 0.986, P value = 2.38e^−12^).

**Table 3:** Parameter estimates using Quasi-Poisson model

Coefficients	Coefficients	Exp (coefficients)	Std. error	t value	Pr (>|t|)
(Intercept)	1.3620765	3.9012	0.21603	6.305	4.84e^−10^***
Age	−0.0121754	0.988	0.00171	−7.126	2.38e^−12^***
Weight	0.0213740	1.0216	0.00277	7.706	3.98e^−14^***
Initial.CD4	0.0034295	1.0212	0.00042	8.143	1.54e^−15^***
Gender (ref = female)					
Gender male	−0.0144168	0.9856	0.02292	−0.629	0.5296
Residence (rural)					
Urban	0.0412981	1.0422	0.02309	1.789	0.0740
Education (ref. = no edu.)					
Educ. primary	0.0191098	1.0192	0.04796	0.398	0.6904
Educ. secondary	0.0280730	1.02847	0.04215	0.666	0.5056
Educ. tertiary	0.0627961	1.0648	0.04688	1.340	0.1808
Marital status (ref = without part)					
With part	0.0939004	1.09845	0.03196	2.938	0.0034**
Household income (ref = high)					
Low income	−0.4627385	0.62955	0.06061	−7.634	6.71e^−14^***
Middle income	−0.3010270	0.74322	0.04312	−6.982	6.29e^−12^***
Owner of cell phone (ref = with cell phone)					
Without phone	−0.4021422	0.66888	0.04867	−8.263	6.15e^−16^***
Adherence (ref = good)					
Adherence fair	−0.5250367	0.5921	0.0596023	−2.517	0.0120*
Adherence poor	−0.6800367	0.50621	0.0596023	−1.267	<2e^−16^***
Level of exposedness (ref = exposed)					
Not exposed	−0.0968024	0.9077	0.0423628	−2.285	0.0226*
WHO stages (ref = stage 1)					
WHO. Stage stage 2	−0.1039865	0.90521	0.0389829	−2.667	0.0078**
WHO. Stage stage 3	−0.0949728	0.90981	0.0500913	−1.896	0.0058*
WHO. Stage stage 4	−0.1321290	0.87623	0.0572926	−2.306	0.0214*
Marital status* adherence (ref = good adherence and living with partner)					
Living with partner* poor adherence	−2.316	0.09912	0.7866	−0.261	0.461*
Living without partner* fair adherence	−2.415	0.08923	0.7827	−0.234	0.002*
Owner of cell phone* age (ref = with cell phone)					
With cell phone* age	−0.013	0.98721	0.0050	−2.598	0.007*
Marital status* initial CD4 cell count (ref = without partner)					
Living with partner* CD4	0.007	1.007	0.0010	2.651	0.000*

Signif. Codes: 0 ‘***’ 0.001 ‘**’ 0.01 ‘*’ 0.05 ‘.’ 0.1 ‘’ 1

The other predictor variable with significant effect for the variable of interest was found to be initial CD4 cell count (refer to Table 3). For 1 cell/mm^3^ increase of initial CD4 cell count, the log of expected change of CD4 cell count was increased by 0.003 (aRR = 1.02, P value = 1.54e^−15^), keeping the other variables constant. A patient with low household income experienced lower CD4 cell count change as compared to the household with high income (aRR = 0.63, P value = 6.71e^−14^). However, a patient with middle household income, CD4 cell count change was lower than that with high household income. The variable ownership of cell phone had significantly affected CD4 cell count change for 1 month of therapy. Hence, the expected change of CD4 cell count for a patient without cell phone decreased by 43% (aRR = 0.67, P value = 0.0226) as compared to otherwise identical patients with a cell phone. With regard to WHO stages, stages 2 and stage 3 patients’ CD4 changes were lower than that of stage 1 patients. Table 3 also shows significant interaction effects with main effects and the following were significant interaction effects in Table 3.

### Interaction effects of owner of cell phone and age of patients

**Fig. 3:**
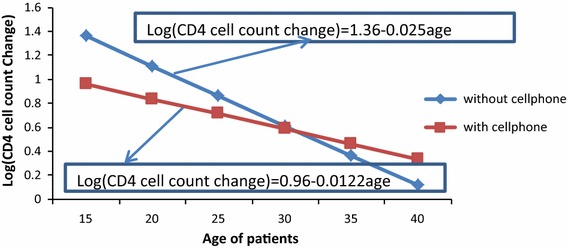
Interaction plot between owner of cell phone and age of patients

Naturally, as age of a patient increases, CD4 cell count decreases, but the decreasing rate of those patients with cell phone was less likely than that of patients without cell phone (aRR = 0.987, P value = 0.007) (refer to Table 3). Figure 3 indicates that the decreasing rate of patients with owner of cell phone is less likely as comapred to those patients without cell phone.

### Interaction between adherence and marital status

**Fig. 4:**
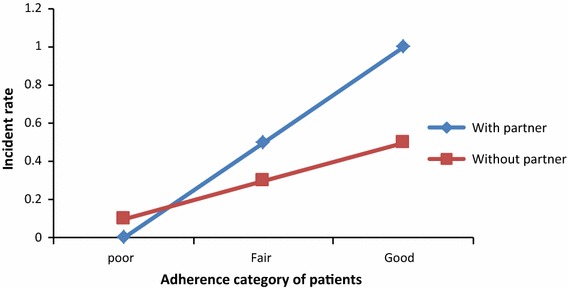
Interaction plot between adherence and marital status of patients

The log of CD4 cell count change for patients with poor and fair adherence living without partners decreased by 2.316 and 2.415, respectively as compared to patients with good adherence living with their partners (aRR = 0.099, P value = 0.003 for poor adherence) and (aRR = 0.089, P value = 0.002 for fair adherent patients) (see Table 3). Figure 4 shows that the incident rate of CD4 cell count change for patients living with partners was by far better than those patients living without their partners.

### Interaction effects of marital status and initial CD4 cell count

**Fig. 5:**
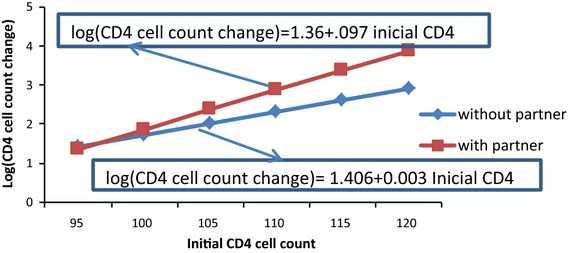
Interaction effect between initial CD4 and marital status of patients

Another significant interaction effect on CD4 cell count change based on 1 month therapy was marital status with initial CD4 cell count. In this 1 month therapy, CD4 cell count change appreciated as initial CD4 count increased, but it was more accelerated for patients living with partners (refer to Fig. 5).

## Discussions

In a month of therapy, CD4 cell count change was highly affected by age, weight, initial CD4 cell count, marital status, income, cell phone ownership, adherence, level of exposedness and WHO stages from the main effect and age with owner of cell phone, marital status with adherence and marital status with initial CD4 cell count from the interaction effect. In this study, as age of an individual increased, CD4 cell count decreased. This is also supported by previous joint longitudinal study [16]. In adherence category, poor adherent patients who did not properly take their medication on time, lose their CD4 cell count. On the other hand, patients with good adherent, who took pills on time regularly, increased their CD4 cell count. A patient living with his/her partner may be encouraged or reminded to take his/her medication on time and this contributes to increase CD4 cell count. A patient who does not expose the disease to family members may not have good adherence to HAART, since he/she takes pills only when nobody is around; and this leads to reduction of CD4 cell count. Naturally, aged people are less likely to have high CD4 cell count as compared to young people. But the decreasing rate of CD4 cell count as age increases was different for patients having cell phone and without having cell phone. Hence patients with cell phone had less decreasing rate as compared to those patients without cell phone.

The significant result of initial CD4 cell count on current CD4 cell count obtained under this study is consistent with a previous study [27]. Hence, a patient who started HAART with high initial CD4 cell count had high CD4 cell count change. On the other hand, an insignificant result of gender on CD4 cell count change in this study contradicted with previous research [27] and is supported by another research [14]. A significant result for marital status obtained in this study is supported by another previous study [11]. The significant result of WHO stages on CD4 cell count in this study is also supported by previous longitudinal study [11].

## Limitations

One limitation of this study was that the interactions between variables were identified in model fit techniques which were not pre-specified or expected during data collection. Therefore, detail information on why these interactions affect on first month CD4 cell count change was not collected and therefore, the reason for some of these findings cannot be explained. Furthermore, this study focused on first month CD4 cell count change. There was no evidence whether or not the factors that affected the CD4 cell count change in first month therapy can also affect the change of CD4 cell count of longitudinal data for the same cohort. The study also tried to identify special characteristics of HIV positive adults and we should not generalize the result to the whole HIV positive people, since the investigation did not include HIV positive patients whose age were less than 15 years. Hence, the result may not be the same on this issue if we incorporate all HIV positive people whose ages are less than 15 years; and this needs further investigation. Therefore, for researchers who want to study this gap it can be considered as potential for further study.

## Conclusions

Quasi-Poisson regression model was a better fit for the given data, and variables that significantly predict the response variable were identified using this model. The result under this investigation indicated that CD4 cell count change of HIV positive people had been affected by several factors. There should be a special attention and intervention for HIV positive adults, especially for those who had low CD4 cell count change, for pre-treatment counseling and awareness creation. The study also tried to identify a certain group of patients who were with maximum risk of CD4 cell count change and need high intervention for counseling and awareness creation. Hence, we recommend that the Ministry of Health (MOH) give due attention for awareness creation so that patients should expose the disease to family members and adhere to HAART directed by health care service providers on time using the alarm of their cell phone as remembrance.

## Abbreviations

AIC: Akaike information criteria
aRR: adjusted rate ratio
BIC: Bayesian information criteria
CI: confidence interval
CD4: classification determinant four
HAART: highly active anti-retroviral therapy
HIV: human immune deficiency virus
SPSS: Statistical Package for Social Science
WHO: World Health Organization
MLE: maximum likelihood estimator
